# Prospective Study for Comparison of Endoscopic Ultrasound-Guided Tissue Acquisition Using 25- and 22-Gauge Core Biopsy Needles in Solid Pancreatic Masses

**DOI:** 10.1371/journal.pone.0154401

**Published:** 2016-05-05

**Authors:** Se Woo Park, Moon Jae Chung, Sang Hoon Lee, Hee Seung Lee, Hyun Jik Lee, Jeong Yup Park, Seung Woo Park, Si Young Song, Hoguen Kim, Jae Bock Chung, Seungmin Bang

**Affiliations:** 1 Division of Gastroenterology, Department of Internal Medicine, Hallym University Dongtan Sacred Heart Hospital, Hallym University College of Medicine, Gyeonggi-do, Korea; 2 Division of Gastroenterology, Department of Internal Medicine, Severance Hospital, Yonsei University College of Medicine, Seoul, Korea; 3 Department of Pathology and Brain Korea 21 Projects for Medical Science, Yonsei University College of Medicine, Seoul, Korea; Hvidovre Hospital, DENMARK

## Abstract

**Background and Aims:**

Although thicker needles theoretically allow more tissue to be collected, their decreased flexibility can cause mechanical damage to the endoscope, technical failure, and sample blood contamination. The effects of needle gauge on diagnostic outcomes of endoscopic ultrasound-guided fine-needle biopsy (EUS-FNB) of pancreatic mass lesions remain unknown. This study compared procurement rates of histologic cores obtained from EUS-FNB of pancreatic masses using 25- and 22-gauge core biopsy needles.

**Patients and Methods:**

From March 2014 to July 2014, 66 patients with solid pancreatic mass underwent EUS-FNB with both 25- and 22-gauge core biopsy needles. Among them, 10 patients were excluded and thus 56 patients were eligible for the analyses. Needle sequences were randomly assigned, and two passes were made with each needle, consisting of 10 uniform to-and-fro movements on each pass with 10 mL syringe suction. A pathologist blinded to needle sequence evaluated specimens for the presence of histologic core.

**Results:**

The mean patient age was 65.8 ± 9.5 years (range, 44–89 years); 35 patients (62.5%) were men. The mean pancreatic mass size was 35.3 ± 17.1 mm (range 14–122.3 mm). Twenty-eight patients (50%) had tumors at the pancreas head or uncinate process. There were no significant differences in procurement rates of histologic cores between 25-gauge (49/56, 87.5%) and 22-gauge (46/56, 82.1%, *P* = 0.581) needles or diagnostic accuracy using only histologic cores (98% and 95%). There were no technical failures or procedure-related adverse events.

**Conclusions:**

The 25-gauge core biopsy needle could offer acceptable and comparable outcomes regarding diagnostic performance including histologic core procurement rates compared to the 22-gauge core biopsy needle, although the differences were not statistically significant.

**Trial Registration:**

ClinicalTrials.gov NCT01795066

## Introduction

Endoscopic ultrasound-guided fine-needle aspiration (EUS-FNA) is an accurate and sensitive tool for pancreatic solid mass diagnosis, involving few major adverse events [1]. In general, EUS-FNA has an essential role in establishing exact diagnoses and therapeutic strategies for pancreatic solid masses; this method has a high but wide range of sensitivity and specificity (75% to 92% and 82% to 100%, respectively), with an accuracy and incidence of adverse events ranging from 70% to 100% and 0% to 3%, respectively [2–4].

Its usefulness, however, is limited for the following reasons. First, the diagnostic yield of smear cytology with EUS-FNA is largely dependent on the availability of a cytopathologist, and the NPV of the procedure is very low; in other words, FNA findings negative for pancreatic solid masses do not reliably rule out the possibility of pancreatic malignancies [5]. Second, it is hard to discriminate between inflammatory regenerative tissue and well-differentiated neoplasm based only on cytological specimens. In addition, histological analysis of tissue architecture or immunohistochemical staining may be necessary for accurate diagnosis of pancreatic lymphoma or neuroendocrine tumors [6,7]. Furthermore, in the current personalized medicine era, it is becoming increasingly essential to obtain histological tissue for molecular analysis. Therefore, there is a clear need for alternative techniques to improve the diagnostic performance of EUS-guided tissue sampling; an EUS-fine needle biopsy (FNB) device has recently been developed to enable retrieval of optimal core specimens for histologic analysis. Recent studies have reported EUS-FNB to be a feasible, safe, and effective technique for obtaining histological core samples for diagnosis of benign and malignant diseases as well as for staging workup in gastrointestinal malignancies [8,9].

Similar to EUS-FNA, the feasibility and diagnostic yield of EUS-FNB depends on the location, size, and characteristics of target lesions in addition to technical and procedural factors (needle diameter and handling technique, material processing and expertise, and training and interaction between endosonographers and cytopathologists). Furthermore, technical limitations such as mechanical friction of the needle-firing mechanism in larger-caliber needles in the torqued trans-duodenal position, when applied to pancreatic head or uncinated process lesions, may occur with EUS-FNB [10–13]. For this reason, a similarly designed but more flexible FNB device has been developed using a 25-gauge needle platform [14]. Approximately 60% to 70% of diagnosed ductal adenocarcinomas occur in the pancreatic head or uncinated process, underscoring the need for an effective trans-duodenal approach. Furthermore, blood and cellular debris contamination complicate cytopathological interpretation, a common occurrence with the widely used 22-gauge needle.[15] In addition, it is often difficult to penetrate a calcified solid mass with a 22-gauge needle.[15] We hypothesized that a 25-gauge needle would provide an appropriate specimen while more easily penetrating solid pancreatic masses.

However, it is unclear whether the theoretical benefit of 25-gauge needles for EUS-FNB actually results in a higher procurement rate of high-quality histologic core samples compared to 22-gauge needles. Furthermore, no study has evaluated histologic core procurement rates of 25-gauge needles. Therefore, the aim of this study was to compare the procurement rates of histologic cores of the recently developed 25-gauge EUS-FNB device to 22-gauge EUS-FNB for solid pancreatic masses.

## Materials and Methods

### Patients and study design

This study was a prospective study of 66 consecutive patients with pancreatic solid masses who were referred to our medical center for EUS-FNB from 1 March 2014 to 31 July 2014. Patients were eligible for the study based on the following criteria: suspected solid pancreatic masses based on clinical work up and image modality that required cytopathological confirmation. Exclusion criteria included: (a) cystic pancreatic lesions without the evidence of solid component suspected with malignant transformation; (b) hemodynamical instability; (c) severe coagulopathy (international normalized ratio > 1.5 or platelet count < 50,000 cells/cubic millimeter [cmm^3^]); (d) inability to suspend antithrombotic therapy; (e) pregnancy; and (f) refusal to provide informed consent or participate in the study. All patients underwent EUS-FNB under moderate-to-deep balanced propofol sedation based on midazolam according to current guidelines [16]. Patients were monitored for immediate post-procedural adverse events for least 4 hours after completion of the procedure and were followed up for up to 30 days to detect late adverse events; non-surgical patients received clinical follow-ups for at least 6 months.

Written informed consent was obtained from all patients for the procedures performed and their study participation; the protocol for this trial and CONSORT checklist are available as supporting information; see S1 Checklist and S1 Protocol. The study protocol was approved by the institutional review board of the ethics committee of Yonsei University (approval number 4-2012-0856) and registered in a clinical trial database (http://ClinicalTrials.gov Identifier: NCT 01795066). The present trial was registered before recruiting the first participant. The authors confirm that all ongoing and related trials for this study are registered.

### Procedural techniques

All patients underwent EUS-FNB with 22-gauge (Echotip ProCore; Cook Endoscopy Inc, Limerick, Ireland) and 25-gauge (Echotip ProCore; Cook Endoscopy Inc, Limerick, Ireland) needles subsequently for the same pancreatic lesion; the procedures were performed by an experienced echoendoscopist (M.J.C) with a current volume of 750 EUS cases per year, including 150 or more FNA/FNB, using a well-established technique [17]. The FNB device was made of a 140-cm stainless steel with a 5.2 F shaft ending with a beveled tip 4 mm in length within a spiral steel sheath surrounded by a Teflon cover. All procedures were performed with a linear array echoendoscope (Olympus UCT 260, Olympus Co., Tokyo, Japan).

After visualizing the target lesion in the endosonographic plane, the echoendoscopist used color Doppler to identify the optimal position for puncture without intervening vessels between the needle and target lesion. The needle was inserted into the target tissue under EUS guidance via the duodenum for pancreatic head and uncinate masses and via the stomach for pancreatic body and tail masses. Once the lesions were well penetrated with the needle, the stylet was removed; a 10-cm^3^ suction syringe was applied to the needle hub, and 10 uniform back-and-forth movements were performed within the lesion during each needle passage. The needle was then withdrawn into the spiral steel sheath and detached from the echoendoscope. After two individual punctures, the first needle was withdrawn into the catheter and removed; the procedure was repeated in same fashion with the second needle. The FNB needle sequences were randomly assigned in a 1:1 proportion based on a computer-generated random order (22- or 25-gauge first), and two punctures were conducted for each needle respectively. The number of needle passages had previously been decided upon as only two times and was maintained constant. The allocation sequence was concealed using opaque sealed envelopes, and neither the cytopathologist nor the patients were unaware of the treatment allocation.

### Cytopathological analysis using 25- and 22-gauge FNB needles

Tissue samples obtained from the first passage by advancing the stylet within the first needle assembly were immediately smeared onto slides, fixed in a 95% ethanol solution, and stained using the Papanicolaou method for cytological analysis. Tissue samples from the second passage of the first needle were recovered in formalin for histological analysis using the same advancing method. One cytopathologist (K.H), experienced in gastrointestinal cytology and blinded to the type and needle sequence, obtained the tissue samples and viewed all prepared slides. The final cytological results were classified into 4 diagnostic categories (a) positive for malignancy, (b) suspicious for malignancy, (c) negative for malignancy, and (d) non-diagnostic [18].

All samples fixed in formalin for histological analysis were processed in cassette form, embedded in paraffin, and prepared in hematoxylin and eosin for evaluation by the same cytopathologist (K.H) for the presence of a histologic core. If necessary, immunohistochemical staining or other special staining was performed to discriminate between inflammatory regenerative tissue and well-differentiated neoplasm or to confirm neuroendocrine tumors [19]. If a histological core was not obtained, the cytopathologist (K.H) processed the same material as cell-block for cytological analysis.

### Gold-standard reference diagnosis of malignant versus benign disease

A final confirmed diagnosis of benign or malignant disease was according to the following reference methods: (a) definite benign or malignant pathological diagnosis based on analysis of surgically resected specimens from operated patients, (b) disease-specific death, and (c) no signs of disease progression or regression during the 6 month or longer follow-up periods according to clinical course or image modality used for suspected pancreatic inflammation at the time of the procedure.[8,18]

Lesions initially categorized as positive or suspicious for malignancy based on EUS-FNB cytopathology findings, and finally diagnosed as malignancy, were considered true positives; those lesions finally diagnosed as benign disease after clinical follow-up were considered false positives. Similarly, benign histology findings finally diagnosed as benign disease were considered true negatives, while histology findings finally diagnosed as malignancy were considered false negatives. Non-diagnostic results were considered false negatives since the procedure failed to provide a diagnosis.

### Outcome measurements and definitions

The primary outcomes of this study were the procurement rates of histologic cores that the cytopathologist (K.H) considered to be of optimal quality for histological evaluation of the needle gauge used for the EUS-FNB procedure. Histologic core was defined as an architecturally intact piece of tissue sufficient for histologic evaluation of the targeted lesion. The secondary outcomes were defined as diagnostic performance, technical failure, and procedure-related adverse events.

Technical failure was defined as any difficulties, including the inability of the needle to exit from the scope channel, mechanical rupture of the needle, and any needle malfunction that required a new needle. Adverse events were defined as immediate or late bleeding, immediate or late perforation, or any other cardiopulmonary distress during or after EUS-guided sampling as observed by the endosonographer or recovery suite nurse or as reported by patients. Serum amylase levels were checked 4 and 24 hours after the intervention in all patients who underwent EUS-FNB; abdominal computed tomography (CT) scans were performed if abdominal pain occurred or persisted. Acute pancreatitis was defined as serum amylase levels ≥ 3-fold the upper limit of the normal range (>345 g/dL), and for newly developed or worsened pancreatic-type abdominal pain and tenderness with nausea/vomiting, >24 hours after the procedure [20].

### Statistical analysis

To calculate the sample size, we referred to the recent studies[19,21] using the same needle calibers as ours (EUS-FNB with the 25-gauge and 22-gauge needles). In the first study[19] conducted by Bang et al, proportion (%) of optimal histologic core using 22-gauge FNB needle was 70% and proportion (%) of optimal histologic core using 25-gauge FNB needle was 92% in second study[21] cuducted by Iwashita et al. A sample size of 56 pairs achieves 80% power to detect an odds ratio of 4.929 using a two-sided McNemar test with a significance level (alpha) of 0.05. The odds ratio is equivalent to a difference between two paired proportions of 0.276. Assuming a 15% dropout rate, the final sample size was set at 66 pairs.

Descriptive statistics were provided for binary and continuous variables using incidence frequency (%) and mean ± standard deviation and range. McNemar tests were used to compare binary variables, and two-sample *t*-tests were used to compare continuous variables. Sensitivity, specificity, diagnostic accuracy, positive predictive value (PPV), and negative predictive value (NPV) of each needle were calculated based on per protocol analysis. The level of significance was set as P < 0.05. All statistical analyses were performed using PASW Windows, version 18.0.0 (SPSS Inc., Chicago, IL, USA).

## Results

**Fig 1:**
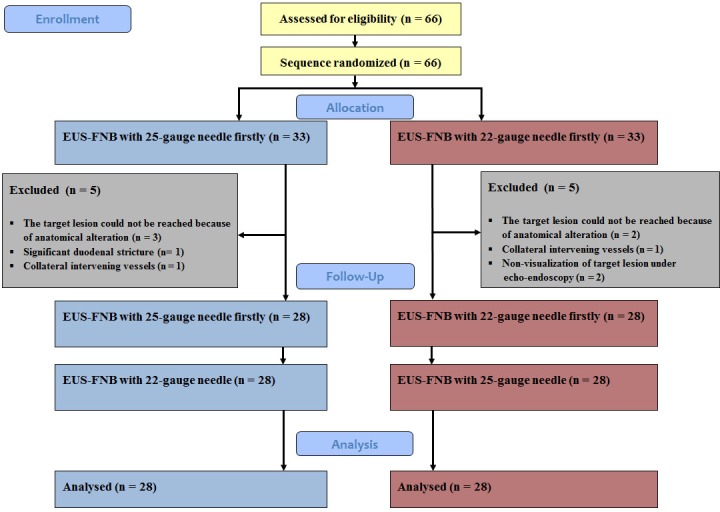
CONSORT Flow diagram of patients throughout the randomized trial. A total of 66 patients were enrolled and underwent initial EUS-FNB with 22-gauge or 25-gauge needle in random sequence. Ten patients were excluded for noncompliance with the scheduled examinations. Among them, the target lesion could not be reached because of anatomical alterations due to previous surgery (e.g., maxillary reconstruction, subtotal gastrectomy with Billroth II reconstruction, or total gastrectomy with Roux en Y reconstruction) in 5 patients, significant duodenal stricture as a result of tumor infiltration in 1 patient, and collateral intervening vessels in 2 patients. Two patients were excluded because the target lesions could not be endosonographically visualized. Thus, a total of 56 patients were eligible for the analyses. In total, 28 patients each initially underwent EUS-FNB with 22-gauge and 25-gauge needles. No patient experienced technical problems (difficult procedure or needle malfunction), after excluding the aforementioned 10 patients from the 66 total patients.

A total of 66 patients were initially enrolled and underwent EUS-FNB with 22- and 25-gauge needles in random sequence (**Fig 1**). Among them, 10 patients were excluded due to an inability to reach target lesions because of anatomical alterations from previous surgery (e.g., maxillary reconstruction, subtotal gastrectomy with Billroth II reconstruction, or total gastrectomy with Roux en Y reconstruction) (n = 5), significant duodenal stricture as a result of tumor infiltration (n = 1), collateral intervening vessels (n = 2), or endosonographical nonvisualization of the target lesion (n = 2). Thus, a total of 56 patients were eligible for the analyses. A total of 28 patients underwent initial EUS-FNB with 22-gauge or 25-gauge needles. No patient experienced technical problems (difficult procedure or needle malfunction), excluding the aforementioned 10 patients among the 66 total patients.

**Table 1**shows demographic and endoscopic characteristics of all patients. A total of 56 patients (35 [62.5%] men; mean age ± standard deviation, 65.8 ± 9.5 years; range, 44–89 years) with solid pancreatic masses were enrolled in a prospective cohort study. Lesions were located in the uncinated process (4 cases, 7.1%), pancreatic head (24 cases, 42.9%), pancreatic body (18 cases, 32.1%), and pancreatic tail (10 cases, 17.9%). The mean size of the pancreatic masses was 35.3 ± 17.1 mm (range 14–122.3 mm). Overall, EUS-FNB was performed via trans-duodenal and trans-gastric approaches in 28 cases (50.0%); the method was technically feasible in all 56 patients enrolled in the study (**Table 1**). There were no procedure-related adverse events in both groups.

**Table 1:** Chracteristics of patients and masses.

Number of patients	56
Age (years ± SD) (range)	65.8 ± 9.5 (44–89)
Sex (Male)	35 (62.5)
Size of lesions (mm ± SD) (range)	35.3 ± 17.1 (14–122.3)
Location (%)	
Head	24 (42.9)
Uncinate process	4 (7.1)
Body	18 (32.1)
Tail	10 (17.9)
Access route	
Trans-gastric	28 (50%)
Trans-duodenal	28 (50%)
Final diagnosis	
Adenocarcinoma	50 (89.2%)
Metastatic renal cell carcinoma	2 (3.6%)
Intra-ductal papillary mucinous neoplasm associated with high grade dysplasia	2 (3.6%)
SCN	1 (1.8%)
AIP	1 (1.8%)

SD, standard deviation; SCN, serous cystadenoma; AIP, autoimmune pancreatitis

Cytological diagnosis results are summarized in **Table 2**. Twenty-six and 23 cases were positive for malignancy using the 25-gauge and 22-gauge needles, respectively. Twenty-five cases were suspicious for malignancy (10 in the head, 2 in the uncinate process, 7 in the body, and 6 in the tail) with the 25-gauge needle, compared to 26 cases (13 in the head, 3 in the uncinate process, 5 in the body, and 5 in the tail) using the 22-gauge needle; in 2 cases (one in the head and one in the body), the specimens were inadequate only in the 22-gauge needle group.

**Table 2:** Cytological diagnostic categories of both needles.

	25 G needle	22 G needle	25 G + 22 G needle
**Diagnostic categories**			
Positive for malignancy (n)	26	23	33
Suspicious for malignancy (n)	25	26	18
Negative for malignancy (n)	5	5	5
Non-diagnostic (n)	0	2	0
**Accuracy** (%)	98%	95%	98%
**Sensitivity** (%)	98%	94%	98%
**Specificity** (%)	100%	100%	100%
**Positive predictive value** (%)	100%	100%	100%
**Negative predictive value** (%)	80%	57%	80%

Fifty (89.2%) lesions were finally diagnosed as primary pancreatic ductal adenocarcinoma, 2 (3.6%) were metastatic renal cell carcinoma and 4 (7.2%) were diagnosed as benign disease (1 as serous cystadenoma, 1 as autoimmune pancreatitis, and 2 as intra-ductal papillary mucinous neoplasm associated with high grade dysplasia) (**Table 1**). The sensitivity, specificity, PPV, and NPV of EUS-FNB with the 25-gauge and 22-gauge needles were 98%, 100%, 100% and 80%, respectively, and 94%, 100%, 100% and 57%, respectively. The diagnostic accuracies of the 25- and 22-gauge needles were 98% and 95%, respectively. If combined, the performance of the 2 needles for each lesion provided sensitivity, specificity, PPV and NPV of 98%, 100%, 100% and 80%, respectively, with an accuracy of 98%.

The 25-gauge needle provided adequate core biopsy tissue samples for histological examination in 49 lesions (87.5%) compared to 46 lesions (82.1%) using 22-gauge needles. While adequate histologic cores were more frequently obtained using the 25-gauge needle compared to the 22-gauge needle, this difference was not significant (87.5% [49/56] vs. 82.1% [46/56]; *P* = 0.599) (**Table 3**). Furthermore, there were no significant differences between the 2 needles regarding the width of adequate histologic cores (0.31 ± 0.20 mm and 0.38 ± 0.28 mm for 25-gauge [n = 49] and 22-gauge [n = 46] needles, respectively; *P* = 0.191) and length of adequate histologic cores (1.67 ± 3.86 mm vs. 2.99 ± 7.99 mm for 25-gauge [n = 49] and 22-gauge [n = 46] needles, respectively; *P* = 0.315). **Table 4**compares the 25-gauge and 22-gauge needles with regard to diagnostic yields using only core biopsy tissue. The diagnostic accuracy, sensitivity, specificity, PPV, and NPV of the 25-gauge and 22-gauge needles were 66.1%, 66%, 66.7%, 94.3% and 19.1%, respectively, and 58.9%, 62%, 33.3%, 88.6% and 9.5%, respectively. The combined performance of the 2 needles for each lesion provided sensitivity, specificity, PPV and NPV of 76%, 66.7%, 95% and 25%, respectively, with an accuracy of 75%.

**Table 3:** Histological comparative data obtained by EUS-FNB using both needles.

	25 G needle	22 G needle	P value
**Procurement of histologic core, n (%)**	49 (87.5)	46 (82.1)	0.581 ^*^
In trans-duodenal approach, n (%)	27 (96.4)	25 (89.3)	0.611
In trans-gastric approach, n (%)	22 (78.6)	21 (75.0)	0.752
Width of histologic core(mm) (mean ± SD)	0.31 ± 0.20	0.38 ± 0.28	0.191
length of histologic core(mm) (mean ± SD)	1.67 ± 3.86	2.99 ± 7.99	0.315

^*^ This value was calculated by McNemar test.

**Table 4:** Histological diagnostic performance of both needles.

	25 G needle	22 G needle	25 G + 22 G needle
**Accuracy**	65.31% [95% CI, 51.11%–77.22%]	60.87% [95% CI, 46.25%–73.77%]	74.07% [95% CI, 60.85%–84.00%]
**Sensitivity**	65.12% [95% CI, 49.07%–78.99%]	62.79% [95% CI, 46.73%–77.02%]	75.00% [95% CI, 60.40%–86.36%]
**Specificity**	66.67% [95% CI, 22.28%–95.67%]	33.33% [95% CI, 0.84%–90.57%]	66.67% [95% CI, 22.28%–95.67%]
**Positive predictive value**	93.33% [95% CI, 77.93%–99.18%]	93.10% [95% CI, 77.23%–99.15%]	94.74% [95% CI, 82.25%–99.36%]
**Negative predictive value**	21.05% [95% CI, 6.05%–45.57%]	5.88% [95% CI, 0.15%–28.69%]	25.00% [95% CI, 7.27%–52.38%]

## Discussion

Although 19-, 22-, and 25-gauge core needles are specifically designed to obtain adequate histological tissue samples, each diameter has technical advantages and limitations. Larger-diameter (especially 19-gauge) needles may provide more tissue; however, they may also be associated with an increased frequency of adverse events, technical failure, or blood and cellular debris contamination. Therefore, the smaller 25-gauge needle was recently developed to improve sampling of adequate histological tissues based on the premise that more flexible, smaller-diameter needles might result in fewer specimens contaminated with blood, more easily penetrate calcified hard masses, and theoretically offer better diagnostic accuracy. This idea was strengthened by several previous analyses of the utility of EUS-FNA in pancreatic masses, using 22- and 25-gauge needle systems [15,22–24].

To our knowledge, this is the first prospective trial of patients with pancreatic masses to compare the technical and diagnostic performance of a newly developed 25-gauge procore biopsy needle specifically designed to acquire histological tissue specimens with 22-gauge needle systems. In our trial, we found that EUS-FNB with a 25-gauge needle system was a technically feasible and comparable tool for procurement of histologic cores from pancreatic masses. Adequate histologic cores were obtained more frequently using the 25-gauge needle (49/56, 87.5%) than using the 22-gauge needle (46/56 82.1%), although the difference was not significant. Moreover, sampling with the 25-gauge needle provided higher diagnostic accuracy (98% vs. 95%), sensitivity (98% vs. 94%), and NPV (80% vs. 57%) than the standard 22-gauge needle, although there were no significant differences between the needles. Furthermore, in our actual practice, 25-gauge needle was more flexible and it was not needed to pull back the echoendoscope up to the body of the stomach in straight position for pushing the thicker needle (22-gauge) out of the working channel. This is a major strength of 25-gauge needle compared to 22-gauge needle. We also found that a 25-gauge needle could easily puncture hard lesions even located at the head of pancreas, particularly those at the uncinate process, which are considered difficult to puncture using the thicker needles. This finding is supported by the results of previous reported studies.[18,23,25]

Iglesias-Garcia et al. [8] reported adequate tissue samples for histological diagnosis of pancreatic masses in 95.7% (45/47) of patients using a 19 guage FNB system, with a high diagnostic accuracy in their pooled cohort study. In general, pushing out the needlefrom the working channel and puncture through trans-duodenal approaches were more difficult; most echoendoscopists prefer to push the needle out of the scope just below the esophago-gastric junction on straight short positions before advancing the scope into the duodenum. To overcome this limitation of large 19-gauge needles, Larghi et al. [9] used a smaller 22-gauge needle in their cohort study to acquire tissue samples from 61 consecutive patients with pancreatic masses. The procedure was technically feasible in all but 1 patient, despite the fact that the puncture was carried out trans-duodenally in 57% of cases. Tissue samples for histological examination were obtained from 55 patients (90.2%; 95%CI: 83–98), core biopsy tissue samples were unable to be retrieved from 5 patients. A correct diagnosis was obtained in all cases for which it was technically possible to obtain adequate samples using FNB. However, there have been no further comparative studies to confirm these findings. Bang et al. [19] compared the diagnostic yield of the new 22-gauge FNB system with that of a 22-gauge FNA assembly, finding no significant differences in the proportion of samples with histologic core tissue samples between the 2 cohorts (100% FNA vs. 83.3%FNB, *P* = 0.26) although optimal histologic core quality was present in 66.7% and 80% of FNA and FNB specimens, respectively (*P* = 0.66).

The major strength of our trial was that FNB was performed on the same pancreatic solid mass lesion with both 22- and 25-gauge needles to allow simultaneous comparison of the effectiveness of the gauges on the same lesion. We considered this direct comparison to be ideal because individual lesions have different characteristic biological features such as fibrosis composition, degree of necrosis, and cell type [26]. Furthermore, a single blinded echoendoscopist conducted FNB to eliminate operator-dependent variability. Additional strength of this study included the random needle sequence and processing of cytological and histological samples by a single blinded cytopathologist.

Interestingly, Sakamoto et al. [27] evaluated the feasibility of a 25-gauge needle compared to a 22-gauge needle in 24 consecutive patients with pancreatic mass who underwent EUS-FNA; they reported a technical success rate of 100% for 25-gauge needles for uncinate process lesions (compared to 33.3% for 22-gauge needles) and pancreatic head lesions (83.3% for 22-gauge needles). In our series, the technical success rates using both 25- and 22-gauge FNB needles for the same lesion were 100% without procedure-related adverse events reported in other studies [8,9,19], although the FNB was technically easier with the 25-gauge needle than the 22-gauge for some difficult locations such as the uncinate process. Our study had comparable results to other studies [23,27,28] using FNA needle systems, which reported that 25-gauge FNA needles tended to result in higher diagnostic accuracy than 22-gauge FNA needles (94% vs. 86%, respectively). However, in respect to diagnostic performance of present study using only adequate histologic specimen, the inferiority of diagnostic accuracy, sensitivity, specificity, PPV and NPV of each needle might be attributed to lower procurement rate of histologic core of each needle (87.5% [49/56] in 25 G needle vs. 82.1% [46/56] in 22 G needle). Truly, the results in Table 4 were based on only adequate histologic specimen instead of cytologic sample including cell block.

Our study had several limitations that might have influenced our final conclusions. First, all procedures were performed only by single experienced echoendoscopists at a single center to minimize variables, as our study aim was to directly compare two different tools used for the same lesion; these conclusions may therefore not be generalizable for all echoendoscopists, and the results should be verified by additional echoendoscopists from additional centers. Second, the sample size was relatively small. Thus, comparisons between groups may have limited reproducibility or generalizability. Third, the criterion-standard reference method used to confirm malignancy or benign disease in our study was not validated. Nevertheless, given the morbidity of pancreatic surgery and the fact that most patients presented with inoperable disease, long-term clinical follow-up and surgical pathology are likely the most reasonable methods for assessment of the performance of EUS-FNB. It is typically believed that “negative for malignancy” in final EUS-FNB pathology reports with definite confirmation for the criterion standard need to involve surgically resected specimens, although it is not always possible for ethical reasons, especially in patients for whom surgery is contraindicated. In our clinical practice, we performed follow-ups for 6 months or more with repeated imaging modalities (EUS, CT, or magnetic resonance imaging). Although not ideal, this practice is a well-accepted reference standard. Fourth, it is possible and reasonable theory that the performing more passes with different size needles in same lesion could result in problems for the collection of adequate tissue, more bloody aspirates and, finally, the possible negative impact of the 2nd needle passes on final cytopathological interpretation.

Although our primary outcome was the procurement rates of histologic cores, its execution had produced disappointing results in regard with diagnostic performance. These results could be attributed to relatively inferior procurement rates of histologic cores in 49 lesions (87.5%) using 25-gauge needles and 46 lesions (82.1%) using 22-gauge needles. Truly, EUS-FNB has no clear advantages over EUS-FNA in terms of overall sensitivity and other diagnostic performance up to this time although EUS-FNB provide a more specific diagnosis in some selected cases. Therefore, we designed our study based on the principle that the collected samples from first pass of each needle allowed only for cytological or cell block analysis in order to assess the optimal diagnosis of patients and the samples from the second pass were processed for histological analysis with or without immune-histochemical staining.

## Conclusions

This study showed that the use of 25-gauge needles for EUS-FNB offered acceptable technical feasibility and comparable procedure-related adverse events compared with 22-gauge needles, although there was no significant differences between both needles regarding diagnostic performance. EUS-FNB might be more easily performed using a 25-gauge needle, especially in particular anatomic sites, and offers at least the same diagnostic performance. Further studies are necessary to validate the optimal needle gauge or combination of needles according to target lesion locations and features.

## Supporting Information

S1 Checklist CONSORT Checklist. (DOCX)

S1 Protocol Study protocol in English. (DOCX)

S2 Protocol Study protocol. (DOCX)
