# Supplementary material for: Prospective Study for Comparison of Endoscopic Ultrasound-Guided Tissue Acquisition Using 25- and 22-Gauge Core Biopsy Needles in Solid Pancreatic Masses
Source: PLoS One. 2016 May 5;11(5):e0154401. doi: 10.1371/journal.pone.0154401 (PMC4858215; doi:10.1371/journal.pone.0154401)
Supplement: S1 Protocol — (DOCX) [file pone.0154401.s002.docx]

**This study protocol was submitted to and approved by the institutional review board of the ethics committee of Yonsei University (approval number 4-2012-0856).**

**1. Study title:** Yield rate for procurement the histologic core with endoscopic ultrasound-guided fine needle biopsy with 25-gauge Ultrasound biopsy needles for solid pancreatic masses

**2. Study director**

**Study director** Se Woo Park, M.D.

Division of Gastroenterology, Department of Internal Medicine,

Severance Hospital, Yonsei University College of Medicine,

Seoul, Korea

**Study director** Moon Jae Chung, M.D.

Division of Gastroenterology, Department of Internal Medicine,

Severance Hospital, Yonsei University College of Medicine,

Seoul, Korea

**Study director** Seung Min Bang, M.D.

Division of Gastroenterology, Department of Internal Medicine,

Severance Hospital, Yonsei University College of Medicine,

Seoul, Korea

**3. Scientific Background of study**

Endoscopic ultrasound-guided fine-needle aspiration (EUS-FNA) is an accurate and sensitive tool for pancreatic solid mass diagnosis, involving few major adverse events [1]. In general, EUS-FNA has an essential role in establishing exact diagnoses and therapeutic strategies for pancreatic solid masses; this method has a high but wide range of sensitivity and specificity (75% to 92% and 82% to 100%, respectively), with an accuracy and incidence of adverse events ranging from 70% to 100% and 0% to 3%, respectively [2-4].

Its usefulness, however, is limited for the following reasons. First, the diagnostic yield of smear cytology with EUS-FNA is largely dependent on the availability of a cytopathologist, and the NPV of the procedure is very low; in other words, FNA findings negative for pancreatic solid masses do not reliably rule out the possibility of pancreatic malignancies [5]. Second, it is hard to discriminate between inflammatory regenerative tissue and well-differentiated neoplasm based only on cytological specimens. In addition, histological analysis of tissue architecture or immunohistochemical staining may be necessary for accurate diagnosis of pancreatic lymphoma or neuroendocrine tumors [6,7]. Furthermore, in the current personalized medicine era, it is becoming increasingly essential to obtain histological tissue for molecular analysis. Therefore, there is a clear need for alternative techniques to improve the diagnostic performance of EUS-guided tissue sampling; an EUS-fine needle biopsy (FNB) device has recently been developed to enable retrieval of optimal core specimens for histologic analysis. Recent studies have reported EUS-FNB to be a feasible, safe, and effective technique for obtaining histological core samples for diagnosis of benign and malignant diseases as well as for staging workup in gastrointestinal malignancies [8,9].

Similar to EUS-FNA the feasibility and diagnostic yield of EUS-FNB depends on the location, size, and characteristics of target lesions in addition to technical and procedural factors (needle diameter and handling technique, material processing and expertise, and training and interaction between endosonographers and cytopathologists). Furthermore, technical limitations such as mechanical friction of the needle-firing mechanism in larger-caliber needles in the torqued trans-duodenal position, when applied to pancreatic head or uncinated process lesions, may occur with EUS-FNB [10-13]. For this reason, a similarly designed but more flexible FNB device has been developed using a 25-gauge needle platform [14]. Approximately 60% to 70% of diagnosed ductal adenocarcinomas occur in the pancreatic head or uncinated process, underscoring the need for an effective trans-duodenal approach. Furthermore, blood and cellular debris contamination complicate cytopathological interpretation, a common occurrence with the widely used 22-gauge needle.[15] In addition, it is often difficult to penetrate a calcified solid mass with a 22-gauge needle.[15] We hypothesized that a 25-gauge needle would provide an appropriate specimen while more easily penetrating solid pancreatic masses.

However, it is unclear whether the theoretical benefit of 25-gauge needles for EUS-FNB actually results in a higher procurement rate of high-quality histologic core samples compared to 22-gauge needles. Furthermore, no study has evaluated histologic core procurement rates of 25-gauge needles. Therefore, the aim of this study was to compare the efficacy of the recently developed 25-gauge EUS-FNB device to 22-gauge EUS-FNB for obtaining histologic core tissues in order to assess solid pancreatic masses.


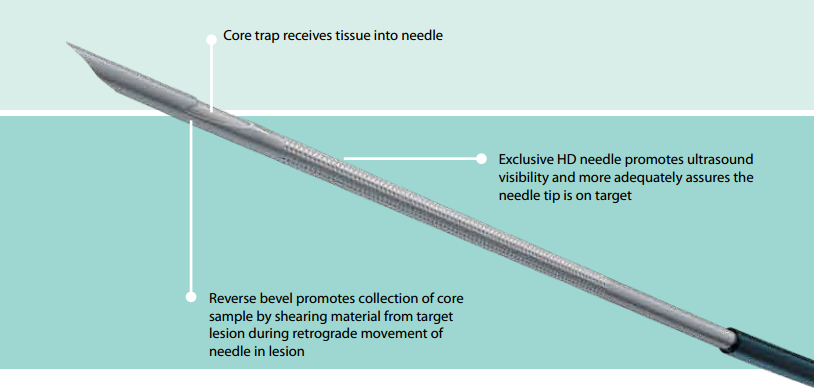


Figure 1. 25-gauge Ultrasound biopsy needle (EchoTip® ProCore™ High Definition Ultrasound Biopsy Needle, Cook Medical Inc, Bloomington, IN)

Figure 2.

**4. Summary of study design**

**Study design:** Single center, prospective, randomized, double-blinded corss-over study

**Study participants:** Planned number: set at 66 pairs

**Study centers:** Severance Hospital, Yonsei University College of Medicine, Seoul, Korea

**Evaluated diagnostic**

**procedure:** Endoscopic ultrasound-guided fine-needle biopsy (EUS-FNB) of pancreatic mass lesions

**5. Sample size calculation**

To calculate the sample size, we referred to the recent studies[19,21] using the same needle calibers as ours (EUS-FNB with the 25-gauge and 22-gauge needles). In the first study[19] conducted by Bang et al, proportion (%) of optimal histologic core using 22-gauge FNB needle was 70% and proportion (%) of optimal histologic core using 25-gauge FNB needle was 92% in second study[21] cuducted by Iwashita et al. A sample size of 56 pairs achieves 80% power to detect an odds ratio of 4.929 using a two-sided McNemar test with a significance level of 0.05. The odds ratio is equivalent to a difference between two paired proportions of 0.276. Assuming a 15% dropout rate, the final sample size was set at 66 pairs.

**6. Inclusion criteria**

suspected solid pancreatic masses based on clinical work up and image modality that required cytopathological confirmation.

**7. Exclusion criteria:**

(a) cystic pancreatic lesions without the evidence of solid component suspected with malignant transformation

(b) hemodynamical instability

(c) severe coagulopathy (international normalized ratio > 1.5 or platelet count < 50,000 cells/cubic millimeter [cmm^3^])

(d) inability to suspend antithrombotic therapy

(e) pregnancy

(f) refusal to provide informed consent or participate in the study

**8. Methods of screening**

Medical record, History, Physical exam, Vital sign, CBC, blood chemistry including serum amylase and lipase, abdominal computed tomography (CT) scans, Pancreato-biliary MR

**9. Standard diagnostic modality of study**

EUS-FNA with aspiration 22-gauge needle has an essential role in establishing exact diagnoses and therapeutic strategies for pancreatic solid masses; this method has a high but wide range of sensitivity and specificity (75% to 92% and 82% to 100%, respectively), with an accuracy and incidence of adverse events ranging from 70% to 100% and 0% to 3%, respectively.

**10. Study procedure**

This study will be a randomized, double-blinded, prospective study of 66 consecutive patients with pancreatic solid masses who will be referred to our medical center for EUS-FNB during 1 year after IRB approval in Severance hospital. Patients will be eligible for the study based on the following criteria: suspected solid pancreatic masses based on clinical work up and image modality that required cytopathological confirmation. Exclusion criteria will include: (a) cystic pancreatic lesions without the evidence of solid component suspected with malignant transformation; (b) hemodynamical instability; (c) severe coagulopathy (international normalized ratio > 1.5 or platelet count < 50,000 cells/cubic millimeter [cmm^3^]); (d) inability to suspend antithrombotic therapy; (e) pregnancy; and (f) refusal to provide informed consent or participate in the study. All patients will undergo EUS-FNB under moderate-to-deep balanced propofol sedation based on midazolam according to current guidelines [16]. Patients will be monitored for immediate post-procedural adverse events for least 4 hours after completion of the procedure and will be followed up for up to 30 days to detect late adverse events; non-surgical patients received clinical follow-ups for at least 6 months.

All patients will undergo EUS-FNB with 22-gauge (Echotip ProCore; Cook Endoscopy Inc, Limerick, Ireland) and 25-gauge (Echotip ProCore; Cook Endoscopy Inc, Limerick, Ireland) needles subsequently for the same pancreatic lesion; the procedures will be performed by an experienced echoendoscopist (M.J.C) with a current volume of 750 EUS cases per year, including 150 or more FNA/FNB, using a well-established technique [17]. The FNB device will be made of a 140-cm stainless steel with a 5.2 F shaft ending with a beveled tip 4 mm in length within a spiral steel sheath surrounded by a Teflon cover. All procedures will be performed with a linear array echoendoscope (Olympus UCT 260, Olympus Co., Tokyo, Japan). After visualizing the target lesion in the endosonographic plane, the echoendoscopist will use color Doppler to identify the optimal position for puncture without intervening vessels between the needle and target lesion. The needle will be inserted into the target tissue under EUS guidance via the duodenum for pancreatic head and uncinate masses and via the stomach for pancreatic body and tail masses. Once the lesions will be well penetrated with the needle, the stylet will be removed; a 10-cm^3^ suction syringe will be applied to the needle hub, and 10 uniform back-and-forth movements will be performed within the lesion during each needle passage. The needle will be then withdrawn into the spiral steel sheath and detached from the echoendoscope. After two individual punctures, the first needle will be withdrawn into the catheter and removed; the procedure will be repeated in same fashion with the second needle. The FNB needle sequences will be randomly assigned in a 1:1 proportion based on a computer-generated random order (22- or 25-gauge first), and two punctures will be conducted for each needle respectively. The allocation sequence will be concealed using opaque sealed envelopes, and neither the cytopathologist nor the patients will be unaware of the treatment allocation.

Tissue samples obtained from the first passage by advancing the stylet within the first needle assembly will be immediately smeared onto slides, fixed in a 95% ethanol solution, and will staine using the Papanicolaou method for cytological analysis. Tissue samples from the second passage of the first needle will be recovered in formalin for histological analysis using the same advancing method. One cytopathologist (K.H), experienced in gastrointestinal cytology and blinded to the type and needle sequence, will obtaine the tissue samples and view all prepared slides. The final cytological results will be classified into 4 diagnostic categories (a) positive for malignancy, (b) suspicious for malignancy, (c) negative for malignancy, and (d) non-diagnostic as Table 1 [18].

A final confirmed diagnosis of benign or malignant disease will be according to the following reference methods: (a) definite benign or malignant pathological diagnosis based on analysis of surgically resected specimens from operated patients, (b) disease-specific death, and (c) no signs of disease progression or regression during the 6 month or longer follow-up periods according to clinical course or image modality used for suspected pancreatic inflammation at the time of the procedure.

**11. Data monitoring plan by study director**

The study directors will keep the data related every 10 cases such as CRF in only restricted computer.

**12. Study goals**

**Primary study goal:**

The procurement rates of histologic cores that the cytopathologist (K.H) considered to be of optimal quality for histological evaluation of the needle gauge

**Secondary study goal:**

Diagnostic performance

Technical failure

Procedure-related adverse events

e.g. Technical failure was defined as any difficulties, including the inability of the needle to exit from the scope channel, mechanical rupture of the needle, and any needle malfunction that required a new needle.

Adverse events were defined as immediate or late bleeding, immediate or late perforation, over-sedation requiring reversal medication, or any other cardiopulmonary distress during or after EUS-guided sampling as observed by the endosonographer or recovery suite nurse or as reported by patients. Acute pancreatitis was defined as serum amylase levels ≥ 3-fold the upper limit of the normal range (>345 g/dL), and for newly developed or worsened pancreatic-type abdominal pain and tenderness with nausea/vomiting, >24 hours after the procedure.

**13. Statistical analysis**

Descriptive statistics were provided for binary and continuous variables using incidence frequency (%) and mean ± standard deviation and range. McNemar tests were used to compare binary variables, and two-sample *t*-tests were used to compare continuous variables. Sensitivity, specificity, diagnostic accuracy, positive predictive value (PPV), and negative predictive value (NPV) of each needle were calculated based on per protocol analysis. Two-sided *P*-values were calculated and significance was accepted at 5%. All statistical analyses were performed using PASW Windows, version 18.0.0 (SPSS Inc., Chicago, IL, USA).

**14. Collection and preservation of specimen from participants**

All samples fixed in formalin for histological analysis will be processed in cassette form, embedded in paraffin, and prepared in hematoxylin and eosin for evaluation by the same cytopathologist (K.H) for the presence of a histologic core like as ordinary sample from other general patients. If necessary, immunohistochemical staining or other special staining will be performed to discriminate between inflammatory regenerative tissue and well-differentiated neoplasm or to confirm neuroendocrine tumors [19]. If a histological core will be not obtained, the cytopathologist (K.H) processed the same material as cell-block for cytological analysis. The participants will ask the results of their specimen, discard of their specimen, or withdrawal from study according to our policy of the institutional review board of the ethics committee of Yonsei University.

**15. Observatory variables**

**Physical exam**

Abdominal distention, tenderness, rebound tenderness

Palpation of abnormal abdominal mass

Weight [ kg]

Height [ cm]

Temperature [ ℃]

Blood pressure [ / mm Hg]

**Image modality (abdominal computed tomography (CT) scans, Pancreato-biliary MR, FDG PET/CT etc.)**

Location of the lesion

Size of the lesion

Characteristics of the lesion

Vascular invasion

Operability

**Fators related intervention**

Access route

Number of passes for diagnosis

Puncture with stylet

Number of to-and-fro movements within the lesion

Use of the stylet to harvest the core sample from the needle

Presence of histologic core

Technical difficulty

**Physical exam after intervention**

New or increased upper abdomen or epigastric pain, back pain, and epigastric tenderness

Temperature

Blood pressure

**Laboratory findings after intervention**

CBC

Routine chemistry including amylase and lipase

**Procedure related complication**

**Presence of histologic core**

**Histological diagnostic categories**

**Final histologic diagnosis**

**Final clinical and pathologic diagnosis as reference**

**Quality of histologic specimens**

**16. Predictable adverse events**

Adverse events will be defined as immediate or late bleeding, immediate or late perforation, over-sedation requiring reversal medication, or any other cardiopulmonary distress during or after EUS-guided sampling as observed by the endosonographer or recovery suite nurse or as reported by patients.

Acute pancreatitis was defined as serum amylase levels ≥ 3-fold the upper limit of the normal range (>345 g/dL), and for newly developed or worsened pancreatic-type abdominal pain and tenderness with nausea/vomiting, >24 hours after the procedure.

**17. Predictable cause of drop out**

The target lesion could not be reached because of anatomical alteration

Significant duodenal stricture

Collateral intervening vessels

Non-visualization of target lesion under echo-endoscopy

**18. Plan of Inter-rim analysis**

None

**19. References**

1. Vilmann P, Jacobsen GK, Henriksen FW, Hancke S (1992) Endoscopic ultrasonography with guided fine needle aspiration biopsy in pancreatic disease. Gastrointest Endosc 38: 172-173.

2. Gan SI, Rajan E, Adler DG, Baron TH, Anderson MA, et al. (2007) Role of EUS. Gastrointest Endosc 66: 425-434.

3. Maluf-Filho F, Dotti CM, Halwan B, Queiros AF, Kupski C, et al. (2009) An evidence-based consensus statement on the role and application of endosonography in clinical practice. Endoscopy 41: 979-987.

4. Kida M (2009) Pancreatic masses. Gastrointest Endosc 69: S102-109.

5. Levy MJ, Wiersema MJ (2002) Endoscopic ultrasound in the diagnosis and staging of pancreatic cancer. Oncology (Williston Park) 16: 29-38, 43; discussion 44, 47-29, 53-26.

6. Ribeiro A, Vazquez-Sequeiros E, Wiersema LM, Wang KK, Clain JE, et al. (2001) EUS-guided fine-needle aspiration combined with flow cytometry and immunocytochemistry in the diagnosis of lymphoma. Gastrointest Endosc 53: 485-491.

7. Mesa H, Stelow EB, Stanley MW, Mallery S, Lai R, et al. (2004) Diagnosis of nonprimary pancreatic neoplasms by endoscopic ultrasound-guided fine-needle aspiration. Diagn Cytopathol 31: 313-318.

8. Iglesias-Garcia J, Poley JW, Larghi A, Giovannini M, Petrone MC, et al. (2011) Feasibility and yield of a new EUS histology needle: results from a multicenter, pooled, cohort study. Gastrointest Endosc 73: 1189-1196.

9. Larghi A, Iglesias-Garcia J, Poley JW, Monges G, Petrone MC, et al. (2013) Feasibility and yield of a novel 22-gauge histology EUS needle in patients with pancreatic masses: a multicenter prospective cohort study. Surg Endosc 27: 3733-3738.

10. Larghi A, Verna EC, Stavropoulos SN, Rotterdam H, Lightdale CJ, et al. (2004) EUS-guided trucut needle biopsies in patients with solid pancreatic masses: a prospective study. Gastrointest Endosc 59: 185-190.

11. Varadarajulu S, Fraig M, Schmulewitz N, Roberts S, Wildi S, et al. (2004) Comparison of EUS-guided 19-gauge Trucut needle biopsy with EUS-guided fine-needle aspiration. Endoscopy 36: 397-401.

12. Wahnschaffe U, Ullrich R, Mayerle J, Lerch MM, Zeitz M, et al. (2009) EUS-guided Trucut needle biopsies as first-line diagnostic method for patients with intestinal or extraintestinal mass lesions. Surg Endosc 23: 2351-2355.

13. Thomas T, Kaye PV, Ragunath K, Aithal G (2009) Efficacy, safety, and predictive factors for a positive yield of EUS-guided Trucut biopsy: a large tertiary referral center experience. Am J Gastroenterol 104: 584-591.

14. Levy MJ, Wiersema MJ (2005) EUS-guided Trucut biopsy. Gastrointest Endosc 62: 417-426.

15. Madhoun MF, Wani SB, Rastogi A, Early D, Gaddam S, et al. (2013) The diagnostic accuracy of 22-gauge and 25-gauge needles in endoscopic ultrasound-guided fine needle aspiration of solid pancreatic lesions: a meta-analysis. Endoscopy 45: 86-92.

16. Cohen LB, Delegge MH, Aisenberg J, Brill JV, Inadomi JM, et al. (2007) AGA Institute review of endoscopic sedation. Gastroenterology 133: 675-701.

17. Irisawa A, Hikichi T, Bhutani MS, Ohira H (2009) Basic technique of FNA. Gastrointest Endosc 69: S125-129.

18. Fabbri C, Polifemo AM, Luigiano C, Cennamo V, Baccarini P, et al. (2011) Endoscopic ultrasound-guided fine needle aspiration with 22- and 25-gauge needles in solid pancreatic masses: a prospective comparative study with randomisation of needle sequence. Dig Liver Dis 43: 647-652.

19. Bang JY, Hebert-Magee S, Trevino J, Ramesh J, Varadarajulu S (2012) Randomized trial comparing the 22-gauge aspiration and 22-gauge biopsy needles for EUS-guided sampling of solid pancreatic mass lesions. Gastrointest Endosc 76: 321-327.

21. Iwashita T, Nakai Y, Samarasena JB, Park do H, Zhang Z, et al. (2013) High single-pass diagnostic yield of a new 25-gauge core biopsy needle for EUS-guided FNA biopsy in solid pancreatic lesions. Gastrointest Endosc 77: 909-915.
