# Supplementary material for: Prospective Study for Comparison of Endoscopic Ultrasound-Guided Tissue Acquisition Using 25- and 22-Gauge Core Biopsy Needles in Solid Pancreatic Masses
Source: PLoS One. 2016 May 5;11(5):e0154401. doi: 10.1371/journal.pone.0154401 (PMC4858215; doi:10.1371/journal.pone.0154401)
Supplement: S2 Protocol — (DOCX) [file pone.0154401.s003.docx]

**1. 연구 제목**

**국문**

췌장 종괴성 병변의 진단을 위하여 25 gauge 조직생검세침을 이용한 내시경초음파 유도하 조직생검에서의 조직획득율: 전향적 연구

**영문**

Yield rate for procurement the histologic core with endoscopic ultrasound-guided fine needle biopsy with 25-gauge Ultrasound biopsy needles for solid pancreatic masses

**2. 연구 목적**

췌장 종괴성 병변에서 새로운 25 gauge 조직생검세침의 조직획들율을 확인하고자 한다.

**3. 연구의 과학적 근거**

세침 흡입술을 이용한 세포학적 검사는 고형 췌장 종괴의 진단과 치료에 있어서 필수적인 검사 방법이다.^1^ 일반적으로 이러한 검사 방법은 높은 민감도와 특이도를 보이며 (민감도 75-92%, 특이도 82-100%), 진단정확도 역시 70-100%로 높게 보고되고 있는 반면 합병증의 발생빈도는 0-3%로 보고하고 있다.^2^ 그러나 이러한 장점에도 불구하고 흡인된 세포만 가지고는 비교적 분화도가 좋은 샘암종과 췌장염에서 관찰되는 염증세포를 구분하기 어려운 경우가 많으며, 또한 림프암종, 신경내분비종양, 자가면역성췌장암에서와 같이 진단을 위해서는 세포학적 검사가 아닌 조직학적 검사가 필요한 경우에는 한계를 보이고 있다. 또한 생물학적 표적 치료 등 개별 맞춤 치료를 위하여 분자생물학적 분석을 위한 조직 획득은 점차 그 중요성이 커지고 있다. 최근 이러한 한계를 극복하고자 조직 획득이 가능한 세침 기구들이 개발되고 있으며, 특히 reverse bevel기술을 이용한 Pro-Core 조직생검세침은 비교적 안전하게 사용될 수 있으며 얻어진 조직의 양 및 질도 충분하다는 보고가 잇따르고 있다 (그림1). 이중 22gauge혹은 이보다 더 굵은 바늘 (19gauge)은 이론상으로는 더 많은 조직을 얻을 수 있을 것이라 생각되지만 실제 임상에서 증명된 바로는 내시경 선단을 빠져나올 때 세침이 가지는 고유한 경도로 인하여 기계적 파손이나 시술 실패 등을 야기할 수 있으며 실제 얻어진 조직에서도 혈액이나 기타 조직에 의한 오염이 문제시되고 있다. 이 밖에도 췌장 두부의 종양에서 조직을 채취하기 위해서는 그림과 같이 위와 십이지장 구부를 지나며 구불어진 내시경을 통해서 세침을 십이지장 너머로 관통 시킬 수 밖에 없는데 (그림2) (Digestive Endoscopy 2007;19(suppl.1):S180-205), 굵은 바늘의 세침의 경우 이러한 굴곡 때문에 세침이 제대로 작동하지 않아 조직 채취가 기술적으로 불가능한 경우가 많다. 췌장선암의 경우, 60-70% 가 췌장의 두부에서 발생하고 있는 것을 고려할 때, 이러한 문제는 향후 해결이 시급한 문제라고 할 수 있다. 따라서 본 연구에서 저자들은 쉬운 조작으로 보다 정확한 조직 획득을 위하여 새롭게 개발된 25 gauge 조직생검세침의 조직 획득율을 살펴 보고자 한다.


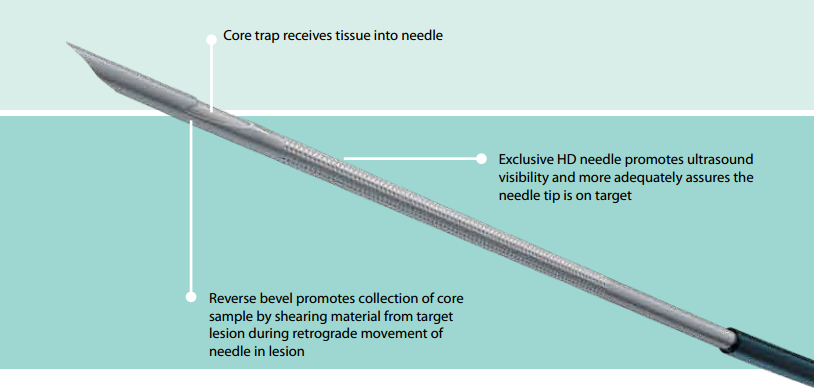


그림1. 25-gauge Ultrasound biopsy needle (EchoTip® ProCore™ High Definition Ultrasound Biopsy Needle, Cook Medical Inc, Bloomington, IN)

그림2.

**4. 연구 설계 개요**

연구심의위원회 승인 후 12개월 간 췌장 고형종괴를 주소로 내원하여 세침흡인검사 혹은 조직 생검이 필요한 연속된 환자를 대상으로 본 연구를 시행한다.

**5. 피험자수 산출근거**

본 연구에서는 각각의 바늘을 이용한 각기 다른 두 선행연구를 참고하였다. Bang등이 보고한 22 gauge 바늘을 사용한 한 연구에서는 조직획득율을 70%로 보고하였으며 Iwashita등이 보고한 25 gauge 바늘을 이용한 한 연구에서는 조직획득율을 92%로 보고하였다. 이 때 본 연구에서는 유의성 0.05 가정하에 two-sided McNemar test를 이용하였을 때 odds ratio 4.929를 추정할 때 80%의 power로 56쌍을 얻을 수 있다. 이 때 odds ratio는 두 쌍의 비율이 0.276의 차이가 동일하다고 가정하였을 때의 값이다. 15%의 탈락율을 가정할 때 최종적으로 66쌍의 피험자가 필요하다.

**6. 선정기준**

임상적 검사 및 CT나 MR 등의 영상학적인 검사를 통하여 췌장 고형종괴를 진단받거나 의심되는 환자에서 내시경초음파 유도하 세침 흡인 세포검사 혹은 조직 생검이 필요한 20세 이상의 자

**7. 제외기준**

1. CT나 MR 등의 영상학적인 검사에서 췌장의 고형종괴가 아닌 낭성 병변인 경우

2. 혈역학적으로 불안정한 자

3. INR>1.5 혹은 혈소판 수 <50,000cell/mm^3^인 자

4. 항응고제 혹은 항혈소판제제의 중단이 어려운자

5. 임산부

6. 본 연구나 동의서에 동의하지 아니한 자 (피험자 중 동의서를 읽을 수 없는 자 포함(예: 문맹, 외국인 등))

**8. 스크리닝 방법**

의무기록, 문진, 신체검진, 생체징후, CBC, blood chemistry including serum amylase and lipase, 복부전산화단층촬영, 췌담도 자기공명영상

**9. 대상질환의 표준 치료 방법**

선형 내시경초음파 유도 하 세침흡인법 혹은 조직생검법은 조직 검사를 위한 다른 방법 특히 복부 초음파 혹은 전산화단층촬영 유도하 경피적 생검 방법에 비하여 몇 가지 장점을 가지고 있다. 첫째, 선형 내시경초음파를 이용한 고해상도 영상을 얻을 수 있으며 둘째, 실시간 도플러 영상을 이용하여 혈관 손상을 피할 수 있으며 셋째, 경십이지장 접근법 등을 통하면 수술 범위 이내로 암세포 오염을 줄일 수 있어 불필요하고 발생 가능한 복강 내 전이를 예방할 수 있다. 이러한 장점들로 인하여 최근에는 선형 내시경초음파 유도하 세침흡인검사가 고형 췌장종괴성 병변의 세포조직학적 진단에 있어 필수적이라 할 수 있겠으며 따라서 일반적으로 22 gauge세침을 이용한 흡인검사가 표준 진단방법으로 받아들여지고 있다.

**10. 연구 방법**

본 연구는 연세대학교 세브란스 병원에 내원하여 임상적 검사 및 CT나 MR같은 영상학적인 검사를 통하여 췌장 고형종괴를 진단받거나 의심되는 환자에서 세침 흡인 세포검사 혹은 조직 생검이 필요한 20세 이상의 자를 대상으로 한다. 이 중에서 1. CT나 MR같은 영상학적인 검사에서 췌장의 고형종괴가 아닌 낭성 병변인 경우, 2. 혈역학적으로 불안정한 자, 3. INR>1.5 혹은 혈소판 수 <50,000cell/mm^3^인 자, 4. 항응고제 혹은 항혈소판제제의 중단이 어려운자, 5. 임산부, 5. 본 연구나 동의서에 동의하지 아니한 자(피험자 중 동의서를 읽을 수 없는 자 포함(예: 문맹, 외국인 등))를 제외한 환자를 대상으로 연구를 진행한다.

연구 기간은 연구심의위원회 승인 후 12개월로 한다. 연구에 포함된 자 중 제외기준에 해당되는 환자를 배제하고 선정된 환자를 대상으로 22guage(EchoTip® Ultra Endoscopic Ultrasound Needle, Cook Medical Inc, Bloomington, IN)와 25guage needle(EchoTip® ProCore™ High Definition Ultrasound Biopsy Needle, Cook Medical Inc, Bloomington, IN) 두 가지 기구를 같은 병변에 대하여 모두 사용한다. 시술은 연간 700례 이상의 초음파 내시경을 시행하며 200례 이상의 세침흡인검사를 시행한 경험이 있는 내시경 시술의사가 진행한다. 모든 시술은 선형 초음파 내시경(Olympus UCT260, Olympus Co., Tokyo, Japan)을 이용하여 진행하며 혈관 흡인을 피하기 위해 도플러(Doppler)를 사용한다. 병변의 위치에 따라서 구상돌기나 췌장두부의 병변은 경십이지장 접근법을, 췌장 체부나 미부의 병변은 경위 접근법으로 시술을 진행한다. 세침이 종괴를 통과한 후에는 음압은 적용하지 않았으며 탐침(Stylet)은 세침을 10-20번 정도 앞뒤로 왕복하는 동안 30초에 걸쳐서 서서히 제거하는 방법을 사용한다. 이 같은 방법으로 한 세침당 2-3회의 통과 과정을 반복할 수 있다. 첫번째 조직생검세침(22guage 세침)을 이용하여 회백색 벌레 모양의 조직이 얻어지면 두번째 세포흡입세침(25guage 세침)을 이용하여 상기 과정을 반복한다. 이렇게 해서 얻어진 두 종류(22-gauge and 25-gauge) 의 조직을 나누어 포르말린 혹은 생리식염수에 고정하여 세침 시행 순서를 모르는 한 조직병리의사에게 판독 의뢰한다.

가장 먼저 core tissue 존재 여부를 먼저 확인한 후 이러한 조직의 질적 평가를 병행한다. 얻어진 조직의 기본 염색(H&E stain)으로 충분한 진단을 얻을 수 있으면 최상(Optimal)으로 분류하고 이것만으로는 불충분하여 면역조직염색이나 다른 특수 염색이 필요한 경우 차선(Suboptimal)로 분류한다.

이렇게 얻어진 검체에 대한 결과는 다음과 같이 4가지 분류하여 보고한다. ^4^

1. Positive for malignancy

2. Suspicious for malignancy

3. Negative for malignancy

4. Non-diagnostic

상기 분류는 아래 table 1에서 열거된 5가지 항목에 대한 점수의 총 합을 기초로 하여 판정한다. 악성세포의 세포충실도가 Fair cellularity로서 2점 이상일 경우 Positive for malignancy로 분류하며 악성세포의 세포충실도가 1점인 경우 Suspicious for malignancy로, 악성세포의 세포충실도가 0점인 경우 Non-diagnostic로 분류한다.

상기 조직학적 진단을 비교할 진단으로 1. 수술을 시행받은 환자에서 얻어진 전체 절제 조직, 2. 수술이 불가능한 환자에서 내시경초음파를 통해 얻어진 조직학적 진단이 악성임을 강력히 시사할 때 다른 영상학적 검사와 임상 경과, 3. 내시경초음파를 통해 얻어진 조직학적 진단에서 악성을 시사할만한 명백한 증거가 없는 상태에서 최소 6개월 이상의 임상 추적 관찰을 기준으로 삼는다. 환자의 기본정보와 임상정보, 시술과 관련된 세부 진행상황 등을 기재하고 병변의 위치와 특성, 시술 관련 합병증 유무 및 조직학적 소견을 기재한다. 성공적 시술은 시술 시 세침의 통과와 회수가 내시경의 파손 없이 용이하게 이루어졌으며 획득된 조직이 육안적으로 확인이 되었음으로 정의한다.

앞서 분류된 세포조직학적 진단의 진위를 판단하기 위하여 수술적 절제술 후 조직병리 소견, 악성췌장질환 의증으로 인한 사망 혹은 6개월 간의 임상 추적관찰 결과와 비교하여 판정하였으며, 6개월 이상의 임상 추적관찰에도 불구하고 병변이 악화 혹은 호전 어떠한 경과도 보이지 않을 시 염증반응으로 최종 정의하였다.

조직생검 세침검사에서 Positive for malignancy 혹은 Suspicious for malignancy로 진단된 자가 기준 진단법에서 악성 질환으로 진단될 시 True positive로, 기준 진단에서 양성 질환으로 진단될 시 False positives(FPs)로 정의하였다. 이와 유사하게 조직생검 세침검사에서 양성질환이었으며 최종 기준진단에서 역시 양성질환일 때 True negatives(TNs)로, 최종 기준진단에서 악성일 때나 non-diagnostic case인 경우 모두 False negatives(FNs)로 정의한다.

합병증은 본 시술로 인하여 임상 경과의 변화가 생겨 의사나 회복실 간호사의 적극적 처치가 필요한 출혈, 천공, 저혈압 혹은 해독제의 필요 등의 경우로 정의한다. 복통을 호소하는 환자에서 혈청 아밀라제가 정상 상한치의 3배이상 상승할 때 시술관련 급성 췌장염으로 정의하며 증상이 지속될 경우 복부 전산화단층촬영을 시행한다.

본 연구의 일차 목표는 25gauge 조직채취 세침을 통하여 적절한 진단적 가치를 가지는 조직을 얻을 수 있는 비율을 확인하는 것이다. 또한 진단 정확도, 기술적 결함 및 합병증의 발생 빈도를 분석한다.

**11. 안전점검자(연구자)에 의한 자료안전모니터링계획:**

자료안전에 관하여 연구책임자(정문재)가 직접 모니터링을 시행한다.

첫 례와 매 10례마다 근거문서와 CRF, 프로토콜 등을 대조하여 자료의 완전성을 보증하고, 피험자의 안전성 자료를 검토한다. 또한 접근이 제한된 컴퓨터에 환자에 대한 모든 자료를 저장한다.

**12. 유효성 평가 항목**

일차 목표

정확한 조직 획득을 위하여 새롭게 개발된 25 gauge 조직생검세침의 조직 획득율(procurement of the histologic core)과 조직 적절도(the sample quality as optimal for histological evaluation)

이차 목표

진단 정확도, 기술적 결함의 발생 정도, 합병증의 발생 빈도

**13. 통계분석방법**

독립 변수가 명칭척도일때 평균치의 검정, T검정, paired T검정 혹은 카이제곱검정이나 로지스틱 회귀분석을 시행하며 명칭 혹은 순위척도일 때 분산분석법을 시행한다.

유의수준이 5%미만일 때 통계적 유의성이 있다고 판단한다.

**14. 연구용 검체의 수집 및 보관**

본 연구에서는 환자로부터 췌장고형종괴의 조직을 수집한다. 본 임상시험을 통해 얻어진 조직은 22gauge 세포흡입세침 및 25gauge 조직생검세침 두 가지로 분류되어 조직병리의사에게 의뢰된다. 즉 25gauge 조직생검세침을 이용하여 얻어진 시험 조직은 22gauge 세포흡입세침을 이용하여 얻어진 조직 혹은 세포와 동일하게 조직학적 진단을 위한 과정을 거치게 되며, 얻어진 조직은 22gauge 세포흡입세침을 통해서 얻어진 조직 혹은 세포들과 마찬가지로 본원의 검사결과에 입력이 되며 추후에도 언제든지 환자의 요청이 있을 시 확인할 수 있다. 무작위 임의 배정 연구가 아니며 또한 추가적인 비용의 발생 없이 일반적인 검체와 동일한 과정을 거치고 결과보고되기 때문에 익명화계획은 없다. 마찬가지로 분석 후 폐기의 과정도 일반 검체와 동일하게 적용된다. 즉 조직은 일반 환자의 조직과 마찬가지로 본원의 규정에 따라 일정기간 보관 후 폐기하게 된다.

**15. 관찰항목**

**이학적 검사**

복부 팽만, 압통, 반발통

종괴성 병변의 촉진

**신체 검사**

Weight [ kg]

Height [ cm]

Temperature [ ℃]

Blood pressure [ / mm Hg]

**영상학적 검사(복부 전산화 단층촬영, 복부 자기공명영상, FDG PET/CT etc.)**

병변의 위치

병변의 크기

병변의 특성

혈관 침윤 여부

Operability

**시술관련지수**

병변의 위치

병변의 크기

병변의 특성

Access route

Number of passes for diagnosis

Puncture with stylet

Number of to-and-fro movements within the lesion

Use of the stylet to harvest the core sample from the needle

Presence of histologic core

Technical difficulty 유무

**신체 검사**

New or increased upper abdomen or epigastric pain, back pain, and epigastric tenderness

Temperature

Blood pressure

**임상검사**

Amylase

Lipase

**Procedure related complication**

**Presence of histologic core**

**Histological diagnostic categories**

**Final histologic diagnosis**

**Final clinical and pathologic diagnosis as reference**

**Quality of histologic specimens**

**16. 예측 부작용 및 주의사항**

시술 중 합병증의 발생빈도는 0-3%로 보고하고 있으며 실제 내시경초음파 유도하 세침검사가 보편화된 최근의 연구들에서 합병증의 발생빈도는 이보다 낮게 보고하고 있다. 25gauge 조직생검세침을 이용할 경우 기존의 22gauge 세포흡입세침으로 검사를 시행받게 되었을 때 발생할 수 있는 합병증의 동일한 범위 내에서 이보다 같거나 드문 확률로 명치 통증, 위장관 출혈, 췌장염 혹은 천공 등이 발생할 수 있다. 따라서 시술 후 지속적인 복통, 복부 팽만, 발열/오한 등이 발생하면 연구자에게 즉시 보고하도록 설명한다.

**17. 임상연구 중지/탈락 기준**

지속적인 투약 및 추적 관찰이 필요하지 않은 연구이며 1회에 한하여 피험자의 동의 하에 검사를 진행하게 된다. 그러나 조직생검을 포함한 검사 후 피험자가 원하다면 연구에서 제외되며 이와 연관된 임상적 자료 및 검체는 즉시 폐기한다.

**18. 중간분석에 대한 계획**

해당없음.

**19. 참고 문헌**

1. Vilmann P, Jacobsen GK, Henriksen FW, Hancke S (1992) Endoscopic ultrasonography with guided fine needle aspiration biopsy in pancreatic disease. Gastrointest Endosc 38: 172-173.

2. Gan SI, Rajan E, Adler DG, Baron TH, Anderson MA, et al. (2007) Role of EUS. Gastrointest Endosc 66: 425-434.

3. Maluf-Filho F, Dotti CM, Halwan B, Queiros AF, Kupski C, et al. (2009) An evidence-based consensus statement on the role and application of endosonography in clinical practice. Endoscopy 41: 979-987.

4. Kida M (2009) Pancreatic masses. Gastrointest Endosc 69: S102-109.

5. Levy MJ, Wiersema MJ (2002) Endoscopic ultrasound in the diagnosis and staging of pancreatic cancer. Oncology (Williston Park) 16: 29-38, 43; discussion 44, 47-29, 53-26.

6. Ribeiro A, Vazquez-Sequeiros E, Wiersema LM, Wang KK, Clain JE, et al. (2001) EUS-guided fine-needle aspiration combined with flow cytometry and immunocytochemistry in the diagnosis of lymphoma. Gastrointest Endosc 53: 485-491.

7. Mesa H, Stelow EB, Stanley MW, Mallery S, Lai R, et al. (2004) Diagnosis of nonprimary pancreatic neoplasms by endoscopic ultrasound-guided fine-needle aspiration. Diagn Cytopathol 31: 313-318.

8. Iglesias-Garcia J, Poley JW, Larghi A, Giovannini M, Petrone MC, et al. (2011) Feasibility and yield of a new EUS histology needle: results from a multicenter, pooled, cohort study. Gastrointest Endosc 73: 1189-1196.

9. Larghi A, Iglesias-Garcia J, Poley JW, Monges G, Petrone MC, et al. (2013) Feasibility and yield of a novel 22-gauge histology EUS needle in patients with pancreatic masses: a multicenter prospective cohort study. Surg Endosc 27: 3733-3738.

10. Larghi A, Verna EC, Stavropoulos SN, Rotterdam H, Lightdale CJ, et al. (2004) EUS-guided trucut needle biopsies in patients with solid pancreatic masses: a prospective study. Gastrointest Endosc 59: 185-190.

11. Varadarajulu S, Fraig M, Schmulewitz N, Roberts S, Wildi S, et al. (2004) Comparison of EUS-guided 19-gauge Trucut needle biopsy with EUS-guided fine-needle aspiration. Endoscopy 36: 397-401.

12. Wahnschaffe U, Ullrich R, Mayerle J, Lerch MM, Zeitz M, et al. (2009) EUS-guided Trucut needle biopsies as first-line diagnostic method for patients with intestinal or extraintestinal mass lesions. Surg Endosc 23: 2351-2355.

13. Thomas T, Kaye PV, Ragunath K, Aithal G (2009) Efficacy, safety, and predictive factors for a positive yield of EUS-guided Trucut biopsy: a large tertiary referral center experience. Am J Gastroenterol 104: 584-591.

14. Levy MJ, Wiersema MJ (2005) EUS-guided Trucut biopsy. Gastrointest Endosc 62: 417-426.

15. Madhoun MF, Wani SB, Rastogi A, Early D, Gaddam S, et al. (2013) The diagnostic accuracy of 22-gauge and 25-gauge needles in endoscopic ultrasound-guided fine needle aspiration of solid pancreatic lesions: a meta-analysis. Endoscopy 45: 86-92.

16. Cohen LB, Delegge MH, Aisenberg J, Brill JV, Inadomi JM, et al. (2007) AGA Institute review of endoscopic sedation. Gastroenterology 133: 675-701.

17. Irisawa A, Hikichi T, Bhutani MS, Ohira H (2009) Basic technique of FNA. Gastrointest Endosc 69: S125-129.

18. Fabbri C, Polifemo AM, Luigiano C, Cennamo V, Baccarini P, et al. (2011) Endoscopic ultrasound-guided fine needle aspiration with 22- and 25-gauge needles in solid pancreatic masses: a prospective comparative study with randomisation of needle sequence. Dig Liver Dis 43: 647-652.

19. Bang JY, Hebert-Magee S, Trevino J, Ramesh J, Varadarajulu S (2012) Randomized trial comparing the 22-gauge aspiration and 22-gauge biopsy needles for EUS-guided sampling of solid pancreatic mass lesions. Gastrointest Endosc 76: 321-327.

21. Iwashita T, Nakai Y, Samarasena JB, Park do H, Zhang Z, et al. (2013) High single-pass diagnostic yield of a new 25-gauge core biopsy needle for EUS-guided FNA biopsy in solid pancreatic lesions. Gastrointest Endosc 77: 909-915.
